# Research trends on chemotherapy induced nausea and vomiting: a bibliometric analysis

**DOI:** 10.3389/fphar.2024.1369442

**Published:** 2024-09-13

**Authors:** Chunhui Ning, Yunzi Yan, Yansong Wang, Rui Li, Wenjie Liu, Linjie Qiu, Lingyun Sun, Yufei Yang

**Affiliations:** ^1^ Xiyuan Hospital, China Academy of Chinese Medical Sciences, Beijing, China; ^2^ Graduate College, Beijing University of Traditional Chinese Medicine, Beijing, China

**Keywords:** chemotherapy induced nausea and vomiting, bibliometric analysis, research trends, hot spots, knowledge mapping analysis

## Abstract

**Background:**

CINV is a frequent adverse response to cancer treatment. There is still much to learn about the pathophysiology and initiating event of CINV, which necessitates continued research despite decades of effort. Identifying the current foci of the complex disease and assessing the scientific impact of pertinent study are made more difficult by the abundance of publications on CINV. Therefore, our goals in this article are to evaluate developments in this field, examine patterns in research domains, and gauge the expansion of CINV research production globally.

**Methods:**

Articles about CINV published between 2012 and 2022 were found by searching the Web of Science Core Collection of Clarivate Analytics. The number of publications over time was visualized using Microsoft Office Excel 2019. CiteSpace and VOSviewer were utilized to create knowledge maps that analyzed collaborations between nations, organizations, and writers. They also presented the history of CINV research and highlighted its current areas of focus.

**Results:**

In this study, 846 papers in all were assessed. Most publications (237, 28.01%) came from the United States. University of Toronto was the most productive institution (34, 4.01%). With 25 articles published, or 2.96% of the total, Aapro Matti published the most. The most frequently published journal was found to be Supportive Care (158, 18.68%). “Palonosetron,” “Moderately emetogenic chemotherapy,” “5-HT3 receptor antagonist,” and “Neurokinin 1 receptor antagonists” were considered the hot topics. It can be seen that the research focus is on the drug treatment of chemotherapy-induced nausea and vomiting.

**Conclusion:**

Through bibliometric analysis, we were able to gain profound insights into CINV research for the first time. Researchers looking to uncover research frontiers and comprehend important information in this discipline may find the study’s findings useful.

## 1 Introduction

As one of the three main cancer treatment methods, chemotherapy is among the most often used and successful approaches, along with surgery and radiation therapy (Marx et al., 2013; Chung et al., 2021). As a systemic therapy, it inhibits the growth of cancer cells, which either spread throughout the body or are eradicated by medication. However, because it can harm normal cells while harming tumor cells, it may have adverse reactions and side effects (Pearce et al., 2017). Moreover, CINV is a frequent adverse response to cancer treatment (Lee et al., 2020). The majority of chemotherapy patients had one or more side effects; exhaustion was the most prevalent side effect, occurring in 80% of cases, followed by nausea and vomiting (48%) and pain (48%) (Henry et al., 2008). In particular, nausea and vomiting require special attention because they can worsen the patient’s quality of life, have an adverse effect on food intake (Marx et al., 2016), and may result in poor adherence to chemotherapy, which can lead to dose reductions or discontinuation, all of which have a significant negative impact on the effectiveness of the treatment. Antitumor medications can typically be categorized into four categories: high, moderate, low, and mild emetic risk during antitumor therapy. According to the guidelines, the incidence of vomiting caused by highly emetogenic chemotherapy (HEC) is more than 90% among patients who do not receive prophylactic antiemetic agents within 24 h of chemotherapy; the incidence for moderately emetogenic chemotherapy (MEC) is 30%–90%; the incidence for low-emetogenic chemotherapy (LEC) is 10%–30%; and the incidence for mildly emetogenic chemotherapy is less than 10% (Herrstedt, 2018). The field of chemotherapy-induced emesis has undergone significant transformation with the introduction of novel antiemetic medicines. The most often used antiemetics for CINV prior to the use of olanzapine include dexamethasone, NK-1R antagonists, and 5-HT3R antagonists (Gupta et al., 2021). It is advised to use dexamethasone, neurokinin-1R antagonists, and 5-HT3R antagonists in conjunction for HEC. Combining 5-HT3R antagonists with neurokinin-1R antagonists is recommended for MEC. A single antiemetic medication, such as a dopamine receptor antagonist, dexamethasone, or a 5HT3 receptor antagonist, may also be taken into consideration for LEC. It is not advisable for people undergoing chemotherapy with a mild emetic regimen to regularly take antiemetic medications (Olver et al., 2017; Herrstedt, 2018). Olanzapine, a medication belonging to the benzodiazepine class, is a second-generation psychotropic used to treat bipolar disorder and schizophrenia (Navari, 2014; Yokoe et al., 2019). Olanzapine has been proposed as an effective antiemetic, and since the early 2000s, the National Comprehensive Cancer Network (NCCN) and the Multinational Association for Supportive Care in Cancer (MASCC) have advocated olanzapine for the prevention and treatment of CINV (Saudemont et al., 2020).

There are still a lot of unanswered questions about CINV despite decades of scientific research, which has drawn a lot of interest from academics. As a result, there has been a steady interest in the condition as a research topic, with numerous publications of literature occurring year. Bibliometric analysis is a method that is typically used to thoroughly expose the research status in a particular topic by measuring and visualizing the qualitative, quantitative, and chronological characteristics associated to diverse fields of research. This helps to highlight significant issues for future research (Guler et al., 2016). The current study’s objective is to use knowledge maps to illustrate the state of CINV research now, as well as its knowledge components, research trends, and growing areas during the past 10 years. This article highlights recent advancements in the topic and gives a summary of the research and scholarly contributions that have been made in it.

## 2 Methods

### 2.1 Search strategy

The Web of Science Core Collection provided the data (WOSCC). The complete search strategy is demonstrated in Supplementary Table S1. On 2 April 2023, we finished all of the retrieval and extraction work to prevent bias resulting from the database’s daily changes. The records that were retrieved were called download_txt and saved as plain text files.

### 2.2 Study selection

**FIGURE 1:**
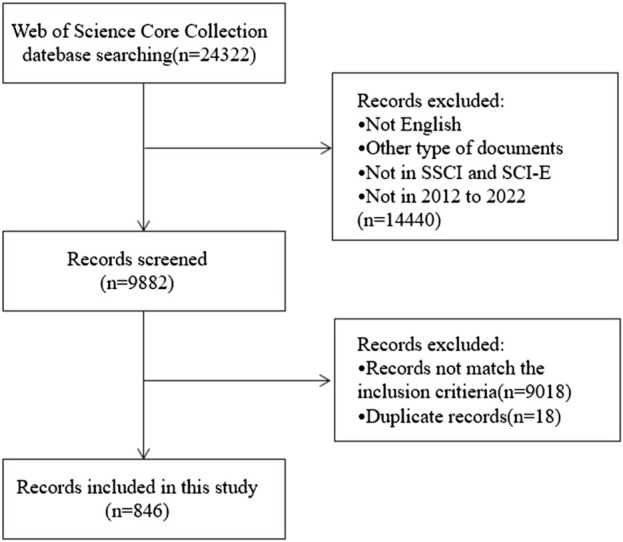
Flowchart of the literature screening process.

Figure 1 depicted the study’s selection criteria and literature screening procedure. Briefly, we entered search terms for an initial search, after then, two researchers went over the papers obtained during the preliminary search and eliminated those that did not meet the specified inclusion requirements as follows: 1) “English” was the only language used for publication; 2) except for letters, comments, reviews, and conference abstracts, the categories of literature included are articles; 3) the source of the publication was the Social Sciences Citation Index (SSCI) and WoSCC Citation Index Expanded (SCI-E) databases; 4) the entire 11-year search period ran from 2012 (1 January 2012) to 2022 (31 December 2022); 5) the subject matter of the chosen article must be chemotherapy-induced nausea and vomiting; it cannot be related to other conditions like pregnancy, disorders of the digestive system, disorders of the nervous system, radiotherapy, etc.

### 2.3 Data analysis

For visualizing the knowledge structure, distribution, and evolution of a particular area, CiteSpace (Chen et al., 2009)—a publicly downloadable software program created by Drexel University professor Chaomei Chen in the USA—is highly popular. There are other resources that provide comprehensive explanations of every indicator that was computed in this study using CiteSpace and VOSviewer (Okhovati and Arshadi, 2021). CiteSpace was used in this study to: 1) create a knowledge map that visualizes collaborations between nations, institutions, and authors; 2) analyse co-citations in references; 3) create a network and cluster map of co-occurring keywords; 4) show the timeline view of co-occurring keywords; and 5) identify references and keywords with strong citation bursts. To visually examine keyword co-occurrence, VOSviewer was utilized. The temporal trends of publications were shown using Microsoft Excel.

## 3 Results

From 1 January 2012, to 31 December 2022, 24,322 records were found. The following factors led to the exclusion of 14,440 records: 1) the literature was not published in English; 2) it was not a research article (reviews, conference abstracts, letters, and ongoing papers); 3) it was not sourced from the WoSCC’s SCI-E or SSCI databases; and 4) the literature was not published in the period of 2012–2022. By reading the complete text or abstract, the remaining 9,882 records were evaluated in more detail. Ultimately, this bibliometric analysis included 846 works that satisfied the inclusion and exclusion criteria after removing 18 duplicate publications (Figure 1). The 846 papers used in this analysis were published in 280 journals, cited 9,842 references from 3,351 publications, were written by 5,029 authors from 1,762 organizations in 61 counties.

### 3.1 Analysis of publications and journals

**FIGURE 2:**
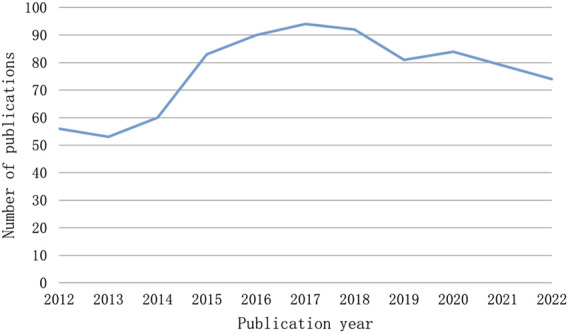
The number of articles published annually in CINV research.

The number of publications over a given time span shows how this field’s research is developing. Figure 2 illustrates the two phases of the research trends, while the quantity of publications on CINV remained relatively constant. With the exception of 2013, the first period from 2012 to 2017 saw an increase in the number of publications. In 2015, at this point, there was an outbreak in CINV research. 2017 saw a peak publishing volume of 94. With the exception of 2020, the publication output in the second stage, which ran from 2017 to 2022, exhibited a declining tendency.

Table 1 lists the top 10 journals for CINV publications published in the past 10 years. The highest production (158, 18.68%) is attributed to supportive care in cancer, with Pediatric Blood and Cancer coming in second (24, 2.84%) and Future Oncology in third (22, 2.60%). Thirty percent of all publications come from the top ten journals in terms of published articles. Annals of Oncology is the most influential journal in the field despite ranking seventh in terms of articles. Out of the 10 journals, it has the highest IF (51.769) and average number of citations (95.33).

**TABLE 1:** The top 10 journals involved in CINV research.

Rank	Journal	Count (%of 846)	Citation	Average citation/Publication	IF (2021)
1	SUPPORTIVE CARE IN CANCER	158 (18.68)	2,730	17.28	3.359
2	PEDIATRIC BLOOD and CANCER	24 (2.84)	424	17.67	3.838
3	FUTURE ONCOLOGY	22 (2.60)	121	5.50	3.674
4	INTERNATIONAL JOURNAL OF CLINICAL ONCOLOGY	16 (1.89)	77	4.81	2.435
5	ANTICANCER RESEARCH	16 (1.89)	284	17.75	2.435
6	ONCOLOGIST	15 (1.77)	164	10.93	5.837
7	ANNALS OF ONCOLOGY	15 (1.77)	1,430	95.33	51.769
8	JOURNAL OF ONCOLOGY PHARMACY PRACTICE	15 (1.77)	52	3.47	1.416
9	BMC CANCER	12 (1.42)	44	3.67	4.638
10	CANCER NURSING	11 (1.30)	103	9.36	2.760

### 3.2 Analysis of the cooperative relationship

**FIGURE 3:**
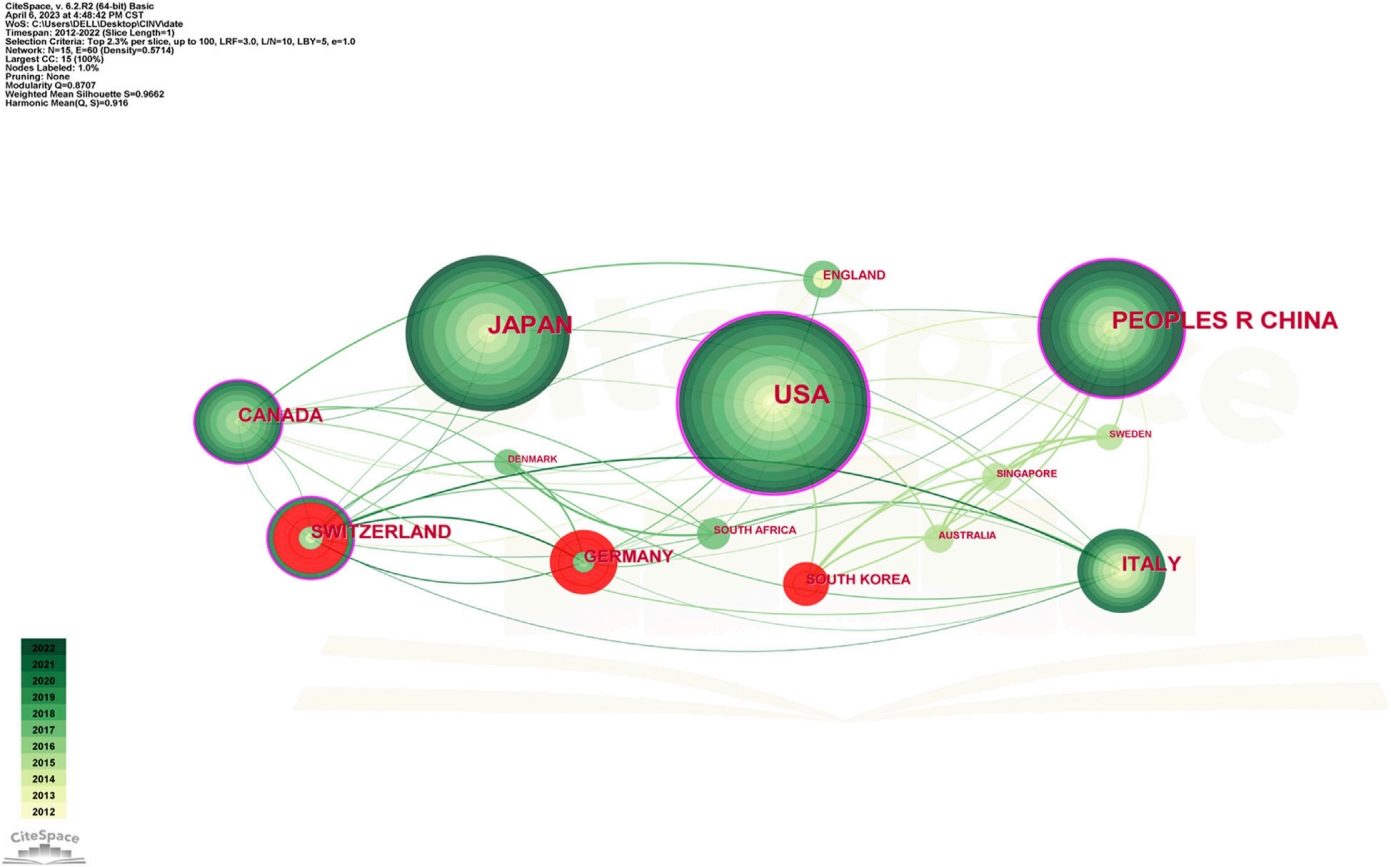
Network of countries and regions engaged in CINV research.

Studies on CINV have been published in 61 different nations and areas. The top ten countries by prolificity are displayed in Table 2. With 237 publications, the United States published the most, followed by Japan (184), China (142), and Switzerland (62). Based on citation counts, the top three countries were the United States (5,169), Switzerland (2,133), and China (1,823). With an average citation/publication of 34.40, Switzerland was highest, followed by Germany (31.72) and England (29.71). Additionally, the United States (0.52), Switzerland (0.24), and China (0.22) were the top three nations in terms of centrality. The collaboration network between these nations is depicted in Figure 3, where each country is represented by a node, the size of which reflects the nation’s publication output. The collaboration between nations is represented by the lines connecting the nodes; the thicker the line, the closer the entities are to cooperating. Different colors are used to identify cooperation groups across nations. There are 31 nations included in this network map, and each country has at least five publications in this field.

**TABLE 2:** The top 10 countries/regions involved in CINV research.

Rank	Country	Count	Citations	Average citation/Publication	Centrality
1	United States	237	5,169	21.81	0.52
2	Japan	184	1797	9.77	0.03
3	China	142	1823	12.84	0.22
4	Swizerland	62	2,133	34.40	0.24
5	Italy	62	1,680	27.10	0.09
6	Canada	58	1,629	28.09	0.03
7	Germany	46	1,459	31.72	0.06
8	South Korea	36	408	11.33	0.00
9	England	31	921	29.71	0.02
10	Australia	27	769	28.48	0.04

Out of all of them, China and the US are probably the two most central and closely connected nations in the network. The United States also has some connections to practically every other nation in the network, with the strongest connections being to Canada, Germany, and Switzerland. China has ties to South Korea, Australia, Canada, and other nations, although it has international links less than the United States. As a result, it is noteworthy that the United States has the largest network of collaboration, extending throughout Asia and Europe.

**FIGURE 4:**
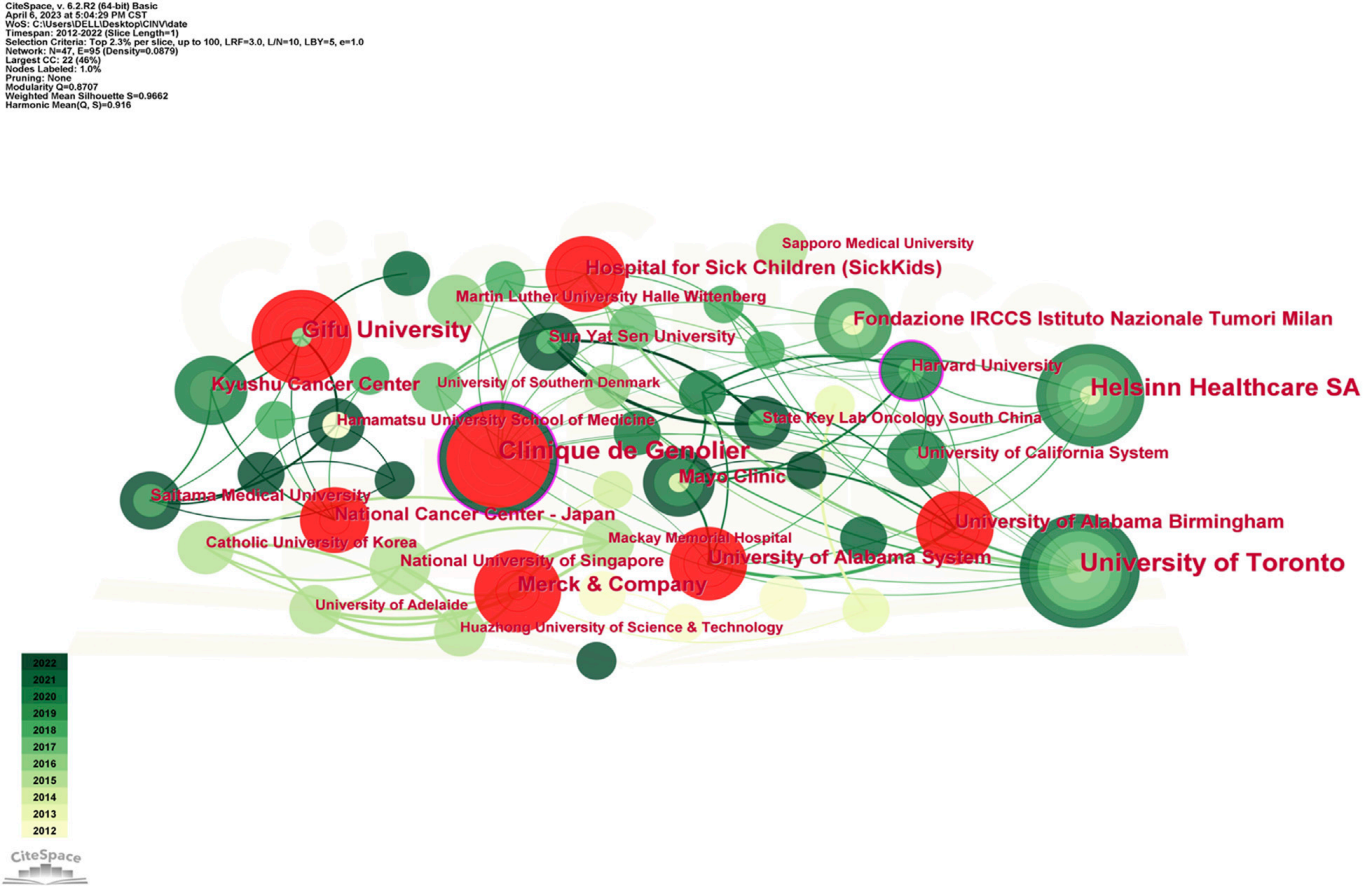
Network of institution engaged in CINV research.

Table 3 displays the top 10 institutions in terms of the quantity of publications each. The most publications were contributed by the University of Toronto (34), with Clinique de Genolier (30), Helsinn Healthcare SA (25), and other institutions following closely behind. University of Toronto also held the record for the most total citations of any institution. The Mayo Clinic was the organization with the greatest average citation/publication. The University of Toronto, the Clinique de Genolier, Merck and Company, and the Fondazione IRCCS Istituto Nazionale Tumori Milan showed the highest degree of centrality (no < 0.1) out of all the institutions. As seen in Figure 4, each institution is represented by each node. A node’s radius grows as it contributes more to the CINV research. The cooperation is represented by the links connecting nodes, whose thicknesses are correlated with the collaboration’s strength. A purple ring indicates a node with a high betweenness centrality rating, and a red ring indicates a burst. Because of their global cooperation, Harvard University and Clinique de Genolier were named as the network’s central countries. The Clinicique de Genolier, for instance, collaborated extensively with the University of Toronto, the University of Alabama System, the Hospital for Sick Children (SickKids), the Fondazione IRCCS Istituto Nazionale Tumori Milan, Harvard University, and other institutions. It was the institution with the widest scientific collaboration.

**TABLE 3:** The top 10 institutions involved in CINV research.

Rank	Institution	Count	Citations	Average Citation/Publication	Centrality
1	University of Toronto	34	838	24.65	0.20
2	Clinique de Genolier	30	661	22.03	0.42
3	Helsinn Healthcare SA	25	545	21.80	0.06
4	Gifu University	23	163	7.09	0.00
5	Merck and Company	22	199	9.05	0.19
6	Hospital for Sick Children (SickKids)	21	471	22.43	0.03
7	Kyushu Cancer Center	19	138	7.26	0.05
8	Fondazione IRCCS Istituto Nazionale Tumori Milan	17	177	10.41	0.21
9	Mayo Clinic	16	445	27.81	0.01
10	National Cancer Center - Japan	16	415	25.94	0.00

**FIGURE 5:**
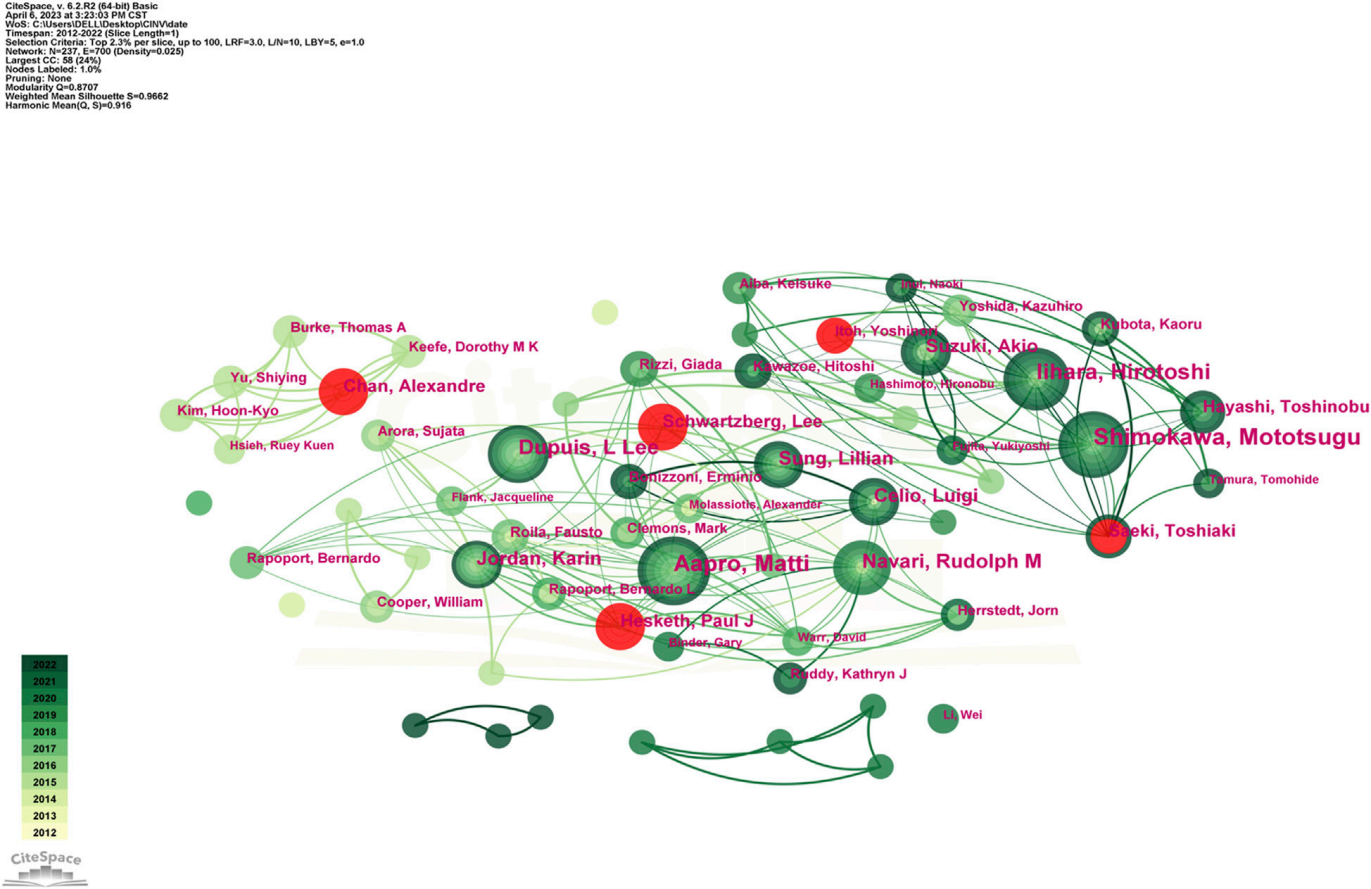
Network of authors engaged in CINV research.

In addition, we evaluated the authors in this study who were the most prolific. With 25 articles, Professor Aapro Matti of the Genolier Cancer Centre was the most productive author. He was followed in output by Shimokawa Mototsugu (23) and Iihara Hirotoshi (20). Likewise, Table 4 shows that Navari Rudolph M had the greatest average citation count (59.29) and total citation count (830). Citespace also carried out the examination of co-authorship (Figure 5). In a similar vein, every node stands for an author, while the lines connecting the nodes show the relationships between authors. The degree of cooperation between the two writers increases with the thickness of the relationship between two nodes. As a result, Aapro Matti Hesketh Paul J, Schnadig Ian D, Rapoport Bernardo L, Clemons Mark, and Molassiotis Alexander are all working closely together. Moreover, there are also noticeable active partnerships amongst Kikkawa Fumitaka, Hayashi Toshinobu, Iihara Hirotoshi, Kitagawa Yuko, and Shimokawa Mototsugu.

**TABLE 4:** The top 10 authors involved in CINV research.

Rank	Author	Count	Citations	Average Citation/Publication
1	Aapro, Matti	25	514	20.56
2	Shimokawa, Mototsugu	23	271	11.78
3	Iihara, Hirotoshi	20	72	3.60
4	Dupuis, L Lee	16	451	28.19
5	Navari, Rudolph M	14	830	59.29
6	Sung, Lillian	14	410	29.29
7	Celio, Luigi	12	174	14.50
8	Schwartzberg, Lee	12	183	15.25
9	Jordan, Karin	12	305	25.42
10	Hayashi, Toshinobu	11	60	5.45

### 3.3 Keyword analysis

Co-citation and citation coupling are the foundations of keyword co-occurrence analysis in bibliometrics (Lee and Su, 2010; Li et al., 2016). The goal is to look at the relationships between keywords that occur frequently in a set of publications that represent popular themes. The more times a phrase appears together, the closer the relationship between those terms is. Keywords were taken from 846 papers for the current investigation. Using a threshold of 10, 242 keywords were found using VOSviewer after unrelated keywords were eliminated and those with similar semantic meanings were combined.

**FIGURE 6:**
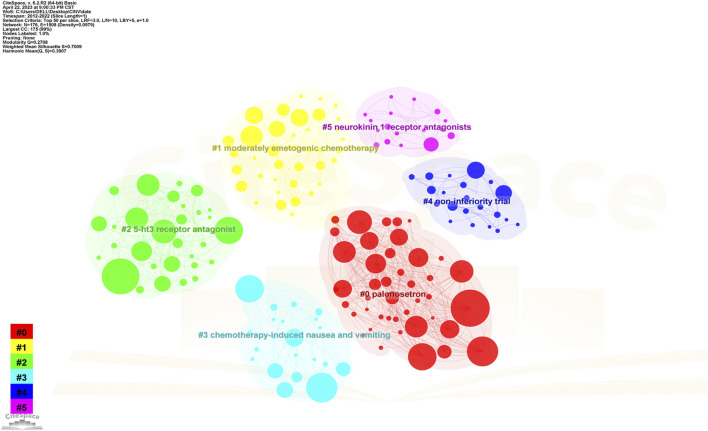
Keyword clustering analysis in CINV research.

**FIGURE 7:**
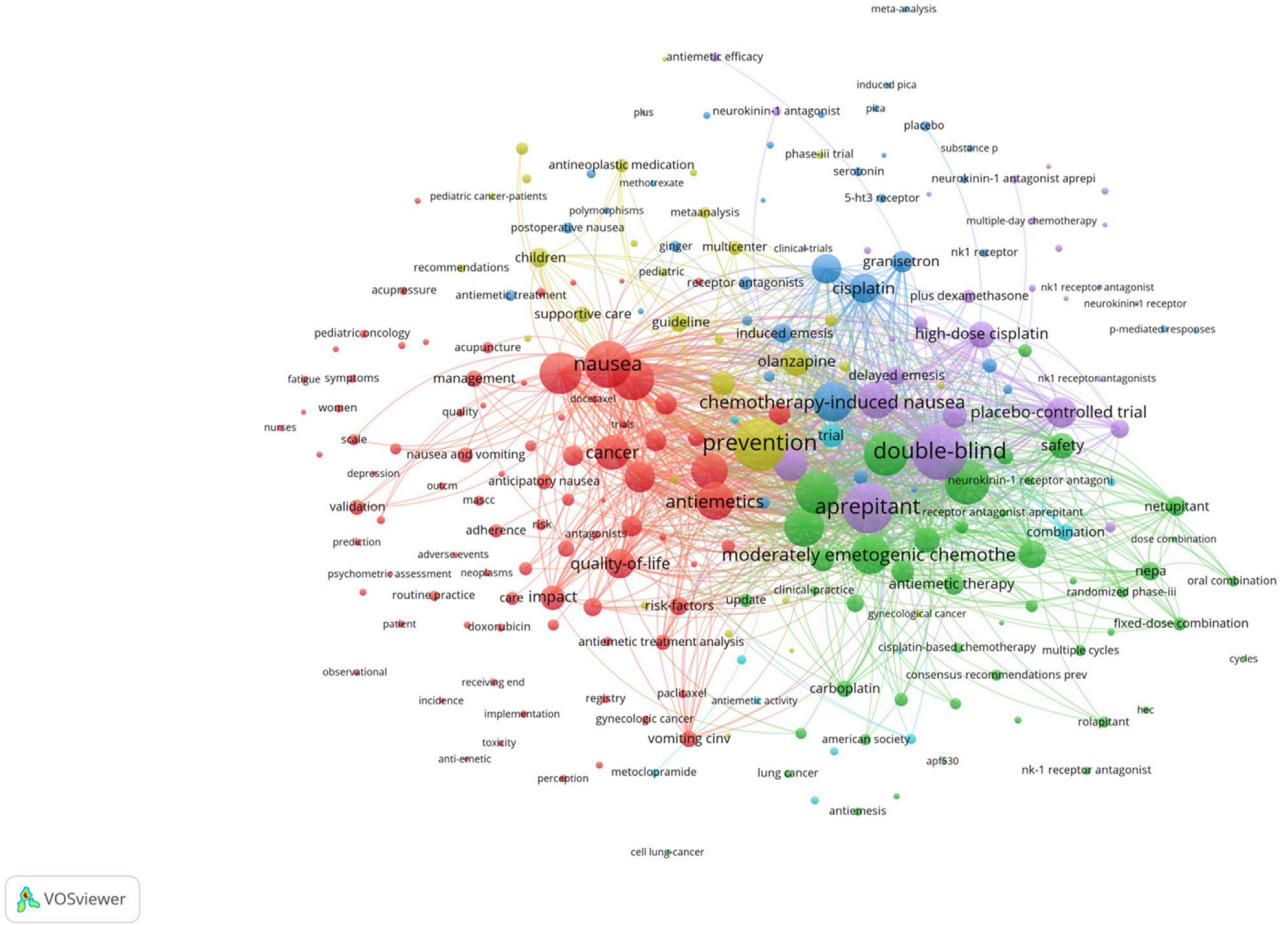
The cooperation network of keywords in CINV research.

The map of keywords with high co-occurrence frequencies that CiteSpace examined is displayed in Figure 6. The keywords were divided into six clusters: the prognostic factors for CINV (light blue cluster), the drug therapy mechanism for CINV (green cluster), clinical trials related to CINV (purple cluster), drug therapy for CINV (dark blue cluster), risk factors for CINV (red cluster), and CINV prevention and support care (yellow cluster). The VOSviewer-generated keyword clustering analysis mapping is displayed in Figure 7. One or more keywords with a particular relationship to each other make create a keyword cluster. The terms “palonosetron” (Cluster 0), “moderately emetogenic chemotherapy” (Cluster 1), “5-HT3 receptor antagonist” (Cluster 2), “chemotherapy-induced nausea and vomiting” (Cluster 3), “non-inferiority trial” (Cluster 4), and “neurokinin 1 receptor antagonists” (Cluster 5) are among the total number of clustering patterns that have been identified. Additionally, these clusters have a large number of lines connecting the nodes, indicating a high co-occurrence rate for keywords.

**FIGURE 8:**
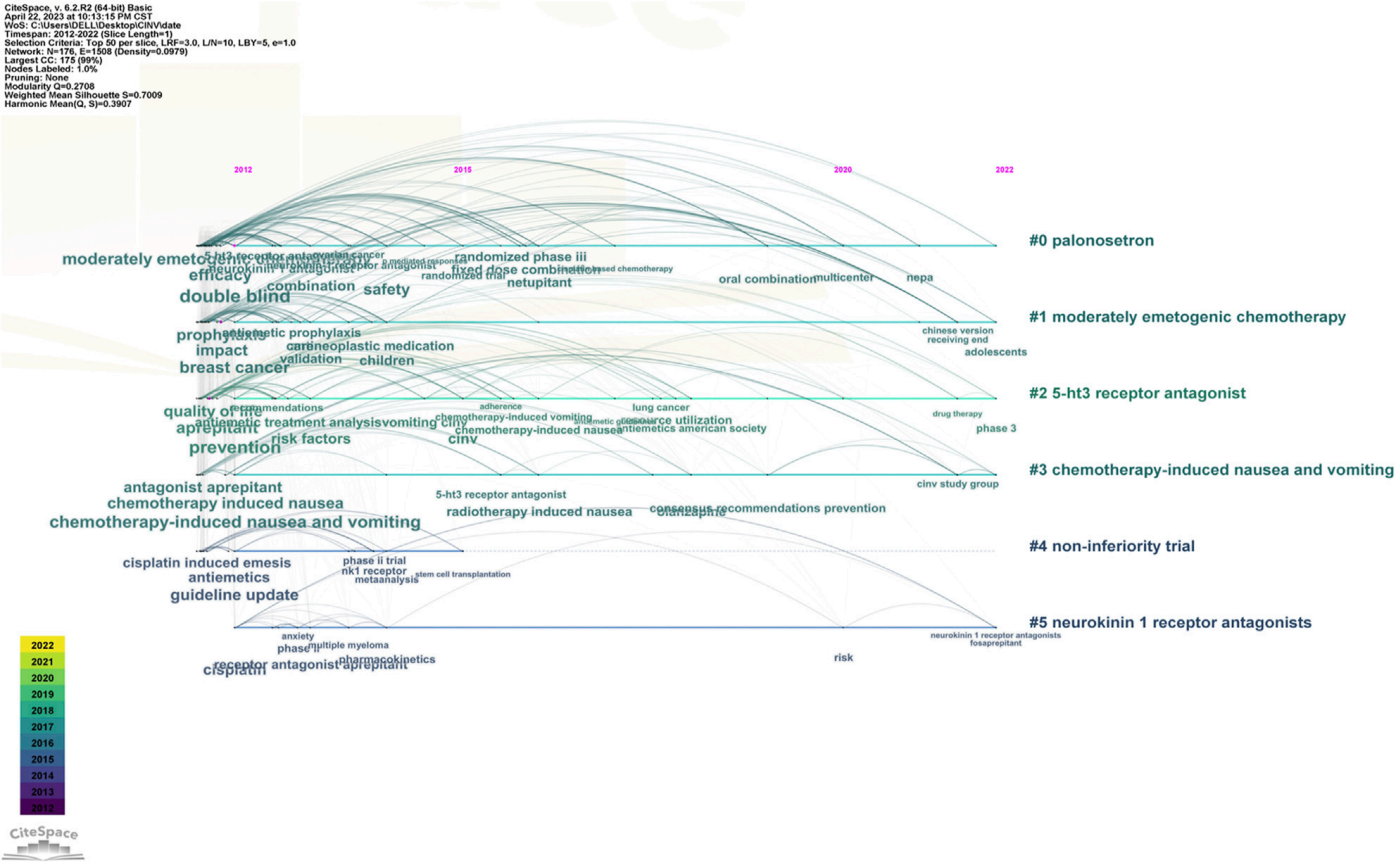
The timeline view of keywords in CINV research.

**FIGURE 9:**
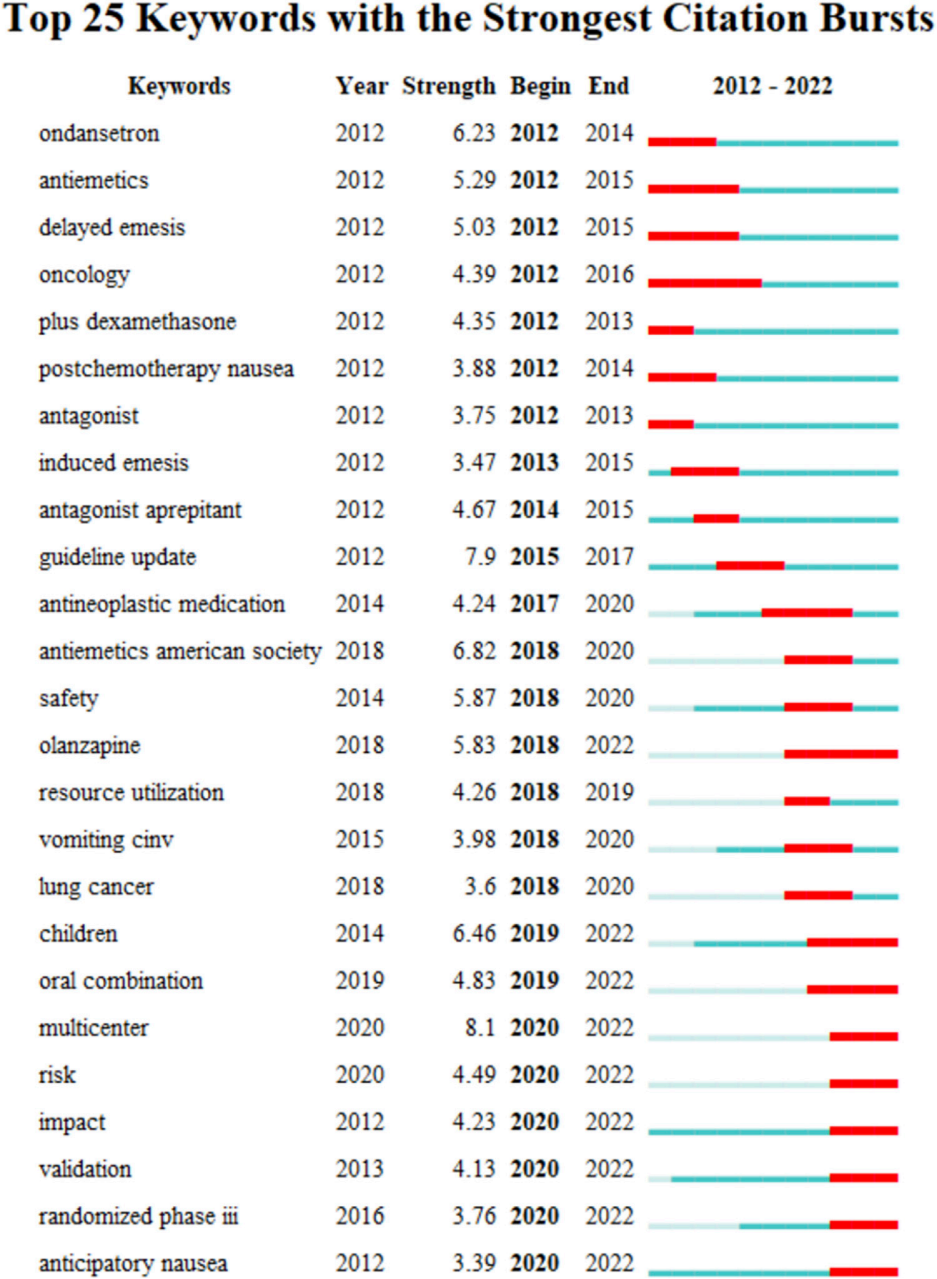
Keywords with strong citation bursts in CINV research.

The keyword co-occurrence timeline view is displayed in Figure 8, which allows us to track the development of research subjects over time. The map contains nodes that stand in for keywords. The links show the co-occurrences of each keyword. The keywords’ chronological sequence reflects the evolution of CINV study themes over time. Burst keywords are also seen to be early warning signs of new trends. Figure 9 displays the keywords in this field with the strongest citation bursts. Year in the figure denotes the first year the keyword appears. The burst’s beginning and finish are represented by the terms begin and end, respectively. When the keywords appear frequently, they are indicated by a red bar, and when they occur infrequently, they are displayed by a blue bar. “Antiemetics American society” has the strongest citation burst out of any of these.

### 3.4 Co-cited references and references burst

490 co-cited references were examined, and Supplementary Table S2 presents the top ten. The main topics covered in these publications, which were thought of as the foundation for information on CINV research, are the epidemiology of CINV, consensus and guidelines for diagnosing and treating the disease, and a sizable randomized controlled placebo clinical trial. Among them, Basch et al. (2011b) published an article, entitled “Antiemetics: American Society of Clinical Oncology Clinical Practice Guideline Update” in Clinical Oncology, which was the most frequently co-cited and ranked first (216), followed by “The oral neurokinin-1 antagonist aprepitant for the prevention of chemotherapy-induced nausea and vomiting: A multinational, randomized, double-blind, placebo-controlled trial in patients receiving high-dose cisplatin—The Aprepitant Protocol 052 Study Group,” written by Hesketh et al. (2003) in Clinical Oncology (165), “Delayed nausea and vomiting continue to reduce patients’ quality of life after highly and moderately emetogenic chemotherapy despite antiemetic treatment” authored by Bloechl-Daum et al. (2006) in Clinical Oncology (159), and “Drug therapy: Chemotherapy-induced nausea and vomiting” published by Hesketh Paul J. (Hesketh, 2008) in Gastroenterology (148). Two of the top ten co-cited articles were published in the esteemed peer-reviewed New England Journal of Medicine (IF 158.5), while half of the top ten were published in the Journal of Clinical Oncology (IF 37.7).

The top 5 co-cited references in Table 5 with the highest betweenness centrality score were thought to be crucial in creating the theoretical foundation of CINV. The study titled “Olanzapine for the Prevention of Chemotherapy-Induced Nausea and Vomiting” (published in the New England Journal of Medicine) had the highest centrality (0.20, 2016). Antiemetics: American Society of Clinical Oncology Clinical Practice Guideline Update (published in the Journal of Clinical Oncology, 0.16, 2017) and The Effect of Guideline-consistent Antiemetic Therapy on Chemotherapy-Induced Nausea and Vomiting (CINV): the Pan European Emesis Registry (PEER) (published in the Annals of Oncology, 0.16, 2014) were next. Top 5 co-cited references focused on 1) updating on guidelines for antiemetic drugs (Basch et al., 2011a; Hesketh et al., 2017); 2) antiemetic drug efficacy (Olanzapine, for example,) in preventing nausea and vomiting in chemotherapy patients (Hesketh et al., 2003; Poli-Bigelli et al., 2003; Warr et al., 2005; Hesketh, 2008; Saito et al., 2009; Navari et al., 2016); 3) effect of guideline-consistent CINV prophylaxis (GCCP) on patient outcomes (Aapro et al., 2012); and 4) impact of CINV on patients’ quality of life (QoL) after emetogenic chemotherapy (Bloechl-Daum et al., 2006).

**TABLE 5:** Top 5 co-cited references with the highest betweenness centrality in CINV research.

Rank	Reference	Centrality	Year
1	Olanzapine for the Prevention of Chemotherapy-Induced Nausea and Vomiting	0.2	2016
2	Antiemetics: American Society of Clinical Oncology Clinical Practice Guideline Update	0.16	2017
2	The effect of guideline-consistent antiemetic therapy on chemotherapy-induced nausea and vomiting (CINV): the Pan European Emesis Registry (PEER)	0.16	2012
3	Olanzapine 5 mg plus standard antiemetic therapy for the prevention of chemotherapy-induced nausea and vomiting (J-FORCE): a multicentre, randomised, double-blind, placebo-controlled, phase 3 trial	0.13	2020
4	Risk factors of chemotherapy-induced nausea and vomiting: Index for personalized antiemetic prophylaxis	0.11	2013
4	2016 updated MASCC/ESMO consensus recommendations: prevention of nausea and vomiting following multiple-day chemotherapy, high-dose chemotherapy, and breakthrough nausea and vomiting	0.11	2017
5	Antiemetic Guideline Consistency and Incidence of Chemotherapy-Induced Nausea and Vomiting in US Community Oncology Practice: INSPIRE Study	0.09	2014

**FIGURE 10:**
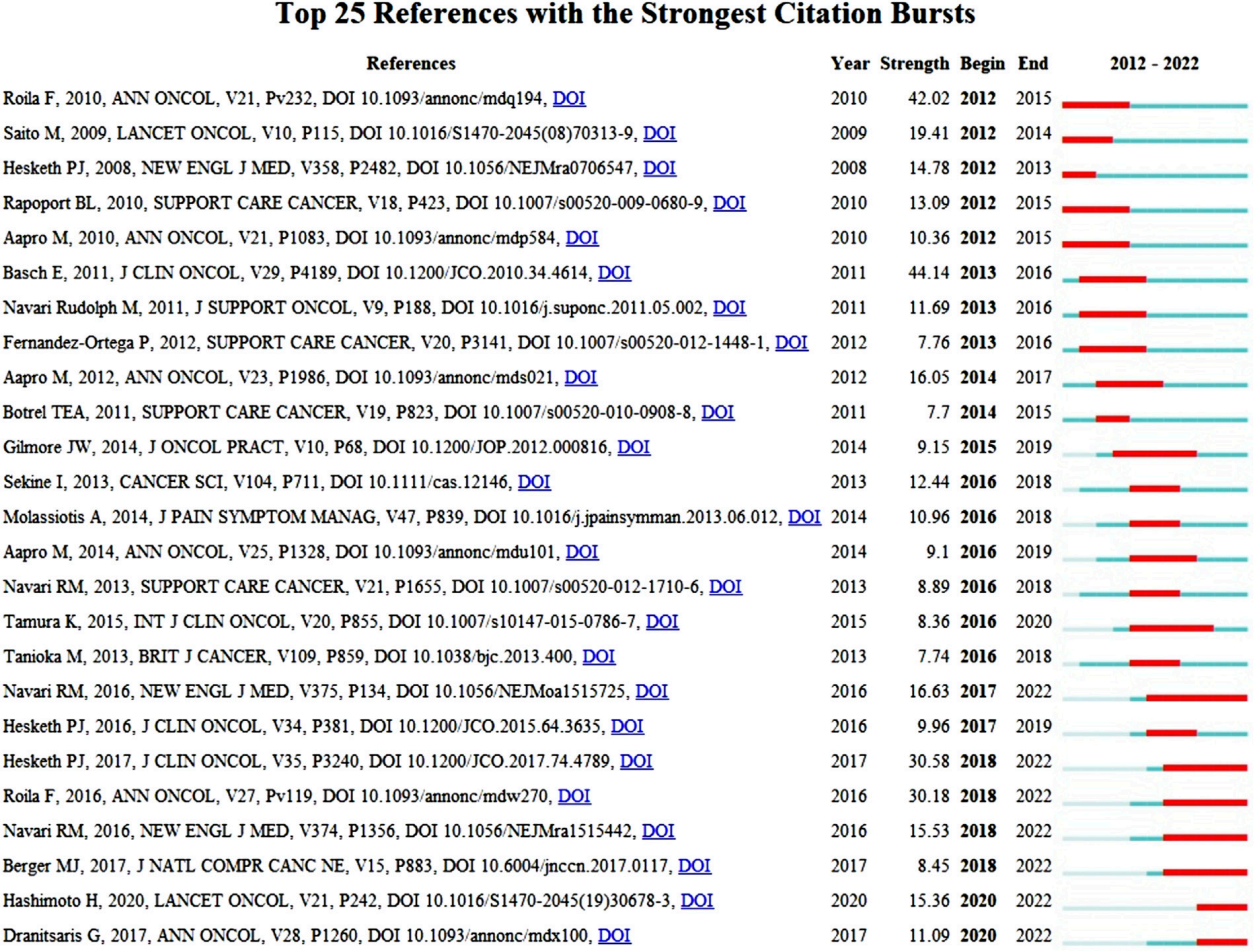
Top 25 references with strong citation bursts in CINV research.

References with bursts of citations can show how a knowledge domain has evolved. Citation bursts are references that academics in a certain discipline pay attention to for a set amount of time. The top 25 references with the strongest citation bursts are shown in Figure 10. For publications connected to CINV, the burst lasted a minimum of 2 years and a maximum of 6 years. The timeline is represented by a blue bar, and the period of time during which a subject is discovered to have a burst is shown by a red segment that shows the beginning year, ending year, and duration of the burst. A higher citation frequency is indicated by a stronger strength. Of these references, 24% (6/25) of the bursts happened in 2016, while 20% (5/25) of the bursts happened in 2012. Remarkably, 60% (15/25) stopped in 2018 or later. The strongest burst (44.14) among the top 25 references occurred for the paper entitled “Antiemetics: American Society of Clinical Oncology Clinical Practice Guideline Update” (Basch et al., 2011b), with a citation burst lasting from 2013 to 2016, followed by “Randomized, double-blind, dose-ranging trial of the oral neurokinin-1 receptor antagonist casopitant mesylate for the prevention of cisplatin-induced nausea and vomiting” (Roila et al., 2009), which was published in Annals of Oncology and exhibited a citation burst from 2012 to 2015 (42.02), and “Antiemetics: American Society of Clinical Oncology Clinical Practice Guideline Update” (Hesketh et al., 2017), which was published in Journal of Clinical Oncology and saw a citation burst from 2018 to 2022 (30.58). Overall, the burst strength of the top 25 references ranged from 7.70 to 44.14, while the most frequent burst duration was 4 years.

## 4 Discussion

### 4.1 General information

CINV research was actively conducted in Europe (Italy, England, Switzerland, and Germany), Asia (China, Japan, and South Korea), North America (the United States and Canada), and Oceania (Australia), as Table 2; Figure 3 demonstrate. A node’s capacity to establish connections with other nodes in the network is referred to as its betweenness centrality. Information flows are more likely to be guided by a source with a high betweenness centrality, demonstrating its versatility in cooperation and potentially revolutionary qualities (Yan and Ding, 2009). Collaboration has been shown to improve the quality of research, and the number of co-authors positively correlates with the impact of citations, particularly in cases when international collaboration is involved (Wuchty et al., 2007). As a result, the United States, Switzerland, China, and Canada played a crucial role in fostering international impact and collaboration in the CINV field.

High-yield educational institutions are primarily found in North America, Asia, and Europe, as Table 3; Figure 4 illustrate. All things considered, the American institutions had wide-ranging international collaboration ties. As an illustration of extensive cross-continental cooperation, the most active institution, University of Toronto, extensively partnered with National University of Singapore, University of South Australia, Ottawa Hospital Research Institute, and Akron Children’s Hospital. But European institutions have few international collaboration links. As a result, the relationship between Clinique de Genolier, which ranked second in scientific output, and institutions such as Ruprecht Karls University Heidelberg, University of Milan, Odense University Hospital, and Helsinn Healthcare SA tended to be limited to be an intra-continent phenomenon.

Scholars from the United States and Japan were clearly the dominant forces, as Table 4; Figure 5 demonstrate. The high ranking of Japanese academics was accompanied by a scarcity of other Asian scholars, particularly those from China and South Korea, which resembled the top institutions’ landscape devoid of any Chinese or Korean institutions. It is plausible that insufficient exchange of resources, ideas, and information hindered CINV research in China and South Korea. Asian nations, including China and South Korea, are therefore urged to pursue global scientific collaboration while raising research productivity, which is directly related to higher research quality and more research capability.

Table 1 indicates that research on CINV has mostly appeared in journals related to supportive care, pediatric hematological cancer, and cancer pharmacy practice. This suggests that CINV treatment is complex and affects patients of nearly all ages.

The quantity of co-citations was typically used to evaluate a study’s impact on the scientific community and its academic success (Moed, 2009). In this domain, journals with a high co-citation rate are called mainstream journals. The majority of high co-citations were discovered in journals with high impact factor (IF), suggesting that CINV research published in prestigious journals has continuously garnered attention.

### 4.2 Knowledge base

Publications that have been referred collectively by other publications are known as co-cited references, and they serve as a kind of knowledge foundation for a specific field of study. The prevention and management of CINV have been covered in great length in a number of articles that were published on the subject between 2012 and 2022. The scientific community has acknowledged these references (Hesketh et al., 2003; Poli-Bigelli et al., 2003; Warr et al., 2005; Bloechl-Daum et al., 2006; Hesketh, 2008; Saito et al., 2009; Basch et al., 2011a; Aapro et al., 2012; Navari, 2014; Hesketh et al., 2017) included in Table 5 as knowledge carriers. This recognition provides a foundation for future research endeavors that aim to generate novel insights. Most of the studies assess the effectiveness of oral medicines as a preventative therapy through randomized controlled trials. The most thoroughly investigated of these medicines are neurokinin-1 (NK1) receptor antagonists, 5-hydroxytryptamine type 3 (5-HT3) receptor antagonists and antipsychotic medication (aprepitant, palonosetron and olanzapine). The corroborative data from Figure 7, which demonstrate that aprepitant forms a key component of knowledge, are also indicative of this argument.

### 4.3 Research frontiers

The research frontiers in this discipline are shown by keywords that exhibit persistent strong citation bursts, as illustrated in Figure 9. The majority of the newly popular subjects are pharmaceuticals like olanzapine.

### 4.4 Medical therapy for CINV

Inhibiting the production of inflammatory mediators, dexamethasone, a corticosteroid often used in 2-, 3-, or 4-drug combinations with other agents, reduces the severity of CINV by acting directly on the solitary tract nucleus (STN), the neurotransmitter 5-HT, and receptor proteins NK1, NK2, etc., while maintaining the patients’ normal physiological functions (Kageyama, 2000; Ho et al., 2004; Suzuki et al., 2004; Dewan et al., 2010). Marco Filetti et al. (2023) collected data from 53 randomized controlled trials (RCTs), which included 22,228 patients with any type of cancer receiving HEC. They then compared various antiemetic regimens to prevent CINV. In terms of complete response, 3- or 4-drug regimens comprising dexamethasone plus NK antagonists, either alone or in combination, produced the highest likelihood of being the most successful regimen. In terms of complete, acute, and delayed response, regimens that combine dexamethasone with a 5-HT3 antagonist have the lowest likelihood of being the most successful regimen (Filetti et al., 2023). When it comes to preventing both acute and delayed CINV in patients receiving HEC and/or MEC, many national guidelines recommend dexamethasone as first-line use in combination with other agents. However, a study conducted by Vardy et al. found that in the week following MEC, patients reported tolerability issues that they linked to dexamethasone, including agitation (27%), increased appetite (19%), weight gain (16%), and acne (15%) (Vardy et al., 2006; Hesketh et al., 2017). These lead us to believe that, when combined with somewhat emetogenic chemotherapy, the negative effects of dexamethasone may exceed its advantages.

In the prevention of CINV, 5-HT3 receptor antagonists (5-HT3 RAs) are essential because they prevent 5-HT3 receptors from binding to serotonin released by enterochromaffin cells in the mucosa of the gastrointestinal (GI) tract in response to chemotherapy (as well as other potentially toxic chemical or mechanical stimuli). This causes the chemoreceptor trigger zone to send a signal to areas within the medulla, which in turn causes increased salivation, respiratory rate, pharyngeal, GI, and abdominal muscle contractions, as well as emesis (Adel, 2017). The structures of the first-generation 5-HT3 RAs, such as ondansetron, dolasetron, granisetron, and tropisetron, were similar to those of serotonin (Hesketh and Gandara, 1991; Lorusso, 2016). The emergence of a second generation of this class of medications was signaled by the invention of palonosetron, a 5-HT3 RA with a structure dissimilar to serotonin. Palonosetron has a longer half-life (of 40 h) for hplasma compared to less than 10 h for old-generation drugs and a binding affinity for the 5-HT3 receptor that is at least thirty times higher *in vitro* than older 5-HT3 Ras (Lorusso, 2016). Palonosetron outperformed older 5HT3 RAs in controlling CINV during the delayed and overall postchemotherapy periods, according to a pooled analysis of four randomized, double-blind, phase III trials comparing palonosetron to ondansetron, dolasetron, and granisetron in the prevention of CINV (Schwartzberg et al., 2014). While 5-HT3 RAs are advised for the first-line prevention of CINV, the medical literature has expressed concerns regarding adverse events (AEs) such as headaches, constipation, elevated ALT, and prolongation of the QT interval, which is linked to severe ventricular arrhythmias (Schwartzberg et al., 2014; Tricco et al., 2016).

By blocking NK1 receptors, NK1 receptor antagonists (NK1 RAs) have been demonstrated to benefit in both acute and delayed CINV by lowering substance *P* activity (Yuan et al., 2016). Findings from a double-blind, randomized, placebo-controlled study conducted in Latin America demonstrated that treatment with aprepitant, the most traditional NK1 RA, in addition to the usual ondansetron and dexamethasone regimen offered better antiemetic protection than standard therapy alone and was generally well tolerated in cancer patients undergoing high-dose cisplatin-based chemotherapy (Poli-Bigelli et al., 2003). NK1 RA netupitant and second-generation 5-HT3 RA palonosetron are combined in a fixed-dose combination called netupitant/palonosetron (NEPA; Akynzeo), which is approved in the EU and the United States for use (in conjunction with dexamethasone) in the prevention of community-acquired pneumonia (CINV) in adults. It is available in oral and, more recently, intravenous (IV) formulations (the IV formulation uses fosnetupitant, a water-soluble prodrug of netupitant). NEPA was compared to aprepitant plus granisetron (an additional combination of an NK1 RA and a 5-HT3 RA) in a phase III non-inferiority trial that was double-blind, randomized, and conducted in Asia. A single oral dose of NEPA was found to be non-inferior to a 3 day regimen of aprepitant with granisetron in this trial. The overall CR rates (primary endpoint) were 73.8% and 72.4%, respectively (between-group difference 1.5%; 95% CI − 4.5% to 7.5%), satisfying the non-inferiority margin of −10% (Zhang et al., 2018).

A member of the benzodiazepine class of psychotropics, olanzapine is a second-generation antipsychotic that was first licensed for the treatment of depression, bipolar disorder, and schizophrenia. However, olanzapine also inhibits dopamine, 5-HT2, and 5-HT3 receptors, which has antiemetic properties (Navari, 2014; Koth and Kolesar, 2017; Filetti et al., 2023). For patients undergoing multiday chemotherapy regimens, olanzapine (5 mg) in combination with fosaprepitant, ondansetron, and dexamethasone proved to be more effective than triple antiemetic treatment alone, according to a randomized, double-blind, placebo-controlled phase 3 trial conducted in 22 hospitals (Zhao et al., 2023). Twelve papers from the database published before 18 April 2021, were eventually included in a meta-analysis by Wang et al. (2021); the study subjects were adult cancer patients receiving HEC or MEC. The purpose of this review was to examine how 10 mg and 5 mg of olanzapine affected the management and prevention of CINV. The findings indicated that individuals with HEC could benefit most from 10 mg of olanzapine, while those with MEC or those without a high risk of CINV might benefit most from 5 mg (Wang et al., 2021). Takako also recruited 153 cisplatin-treated patients for a phase II clinical study. The findings revealed that the incidence of somnolence was 45.5% and 53.3%, respectively, and that the CR for the delayed period was 78% (80% CI: 70.3 – 83.8, *p* = 0.01) in the 10 mg olanzapine group and 86% (80% CI: 79.2–90.7, *p* < 0.001) in the 5 mg olanzapine group (Slimano et al., 2018). Likewise, numerous research investigations have indicated that olanzapine at doses of 10 mg and 5 mg much enhanced delayed vomiting, markedly decreased the frequency and length of nausea in individuals at high risk, and 5 mg olanzapine resulted in reduced somnolence (Slimano et al., 2018; Clemons et al., 2020; Hashimoto et al., 2020; Molassiotis, 2020; Suehiro et al., 2021). Olanzapine is most commonly used to treat nausea and vomiting brought on by chemotherapy drugs; however, the most common side effects are drowsiness and dizziness. These adverse reactions are not immediately apparent, so there is no need to stop using Olanzapine, and there have not yet been any reports of notable extrapyramidal reactions (Tanaka et al., 2019).

Currently, for the medical treatment of nausea and vomiting induced by chemotherapy, such as ondansetron and olanzapine are widely used clinically. However, intensive research has shown that while these drugs exhibit therapeutic effects, they inevitably bring about a series of side effects, which significantly hinder the substantial improvement of patients’ quality of life during chemotherapy. In view of this, future research should focus on exploring and developing complementary and alternative therapies for preventing or treating nausea and vomiting caused by chemotherapy, with the aim of reducing the occurrence of adverse reactions related to drug treatment, thereby enhancing patients’ treatment experience and quality of life in a more comprehensive and safe manner.

## 5 Limitations

There are some restrictions on this study. First off, the study’s analysis is restricted to works released between 2012 and 2022, previous works are not included. Furthermore, the analysis was limited to English-language publications, which may have missed pertinent work that was published in other languages. Additionally, the study’s exclusive reliance on the SCI-E database of WOS limits the variety of literature kinds that may be taken into account. Second, there are some restrictions on the CiteSpace software used for the investigation, like a 500 network size limit, which could affect the analysis’s findings. Despite these drawbacks, the study provides a thorough analysis and synopsis of the accomplishments made in the subject of CINV during the previous several decades, aiding in the comprehension of the field’s current state of development by scholars.

## 6 Conclusion

This work is a comprehensive application of bibliometric and knowledge mapping approaches to analyze the literature on chemotherapy-induced nausea and vomiting (CINV). We used cutting-edge resources like CiteSpace and VOSviewer to improve the scope and depth of our analysis. With the aid of these technologies, we were able to extract a wider range of insightful information from the data. Setting itself apart from conventional evaluations, this work provides a fresh and impartial viewpoint on the state of CINV research. Overall, in the past 10 years, the bibliometric profile of CINV seeks to locate, assess, and illustrate publications concerning qualitative, semi-qualitative, and chronological elements. We also demonstrated that North America and Europe were at the forefront of CINV research with regard to qualitative, quantitative, and collaborative variables. In terms of institutional, regional, and national collaboration, high-yield Asian nations like China and Japan have a dismal track record. To enable future developments in this field of study, this inhibiting trend must shift, and future cooperative activities should be encouraged, supported, and carried out internationally. The publications described NK1 Ras, Olanzapine, and 5-HT3 Ras as potential areas of research for CINV treatments.

## Data Availability

The original contributions presented in the study are included in the article/Supplementary Material, further inquiries can be directed to the corresponding author.
