# Supplementary material for: Research trends on chemotherapy induced nausea and vomiting: a bibliometric analysis
Source: Front Pharmacol. 2024 Sep 13;15:1369442. doi: 10.3389/fphar.2024.1369442 (PMC11427345; doi:10.3389/fphar.2024.1369442)
Supplement: Supplementary file 1 [file DataSheet1.docx]

Supplementary Material

# Supplementary Table

Supplementary Table S1. Search strategy

| **Web of Science Core Collection** |
| --- |
| 1 TS=(neoplasms OR "chemotherapy, adjuvant" OR "consolidation chemotherapy" OR "induction chemotherapy" OR photochemotherapy OR "maintenance chemotherapy" OR "chemotherapy, cancer, regional perfusion" OR "antineoplastic combined chemotherapy protocols" OR electrochemotherapy OR neoplas* OR tumor* OR tumour* OR malignanc* OR cancer* OR chemotherap* OR "antineoplastic combined chemotherapy regimens" OR "antineoplastic chemotherapy protocol*" OR "chemotherapy protocol, antineoplastic" OR "protocol, antineoplastic chemotherapy" OR "cancer chemotherapy protocol*" OR "protocol, cancer chemotherapy" OR "adjuvant chemotherapy" OR "consolidation chemotherapies" OR "chemotherapy, consolidation" OR "regional perfusion antineoplastic chemotherapy" OR "isolation perfusion cancer chemotherapy" OR "cancer chemotherapy, regional perfusion" OR "perfusion cancer chemotherapy, regional" OR "regional perfusion cancer chemotherapy" OR electrochemotherapies OR "chemotherapy, induction" OR "maintenance chemotherapies" OR photochemotherapies OR "chemotherapy induced nausea and vomiting" OR "chemotherapy-induced nausea and vomiting" OR CINV)  2 TS=( nausea OR vomiting OR emetics OR Antiemetics OR emesis OR emetogenic OR emetogenicity OR nausea OR nauseous OR vomit* OR emetic* OR regurgit*)  3 #1AND#2 |

Supplementary Table S2. Top 10 co-cited references in CINV research.

| Rank | Reference | Author | Journal | Year | Citation | Impact Factor |
| --- | --- | --- | --- | --- | --- | --- |
| 1 | Antiemetics: American Society of Clinical Oncology Clinical Practice Guideline Update | Basch Ethan | JOURNAL OF CLINICAL ONCOLOGY | 2011 | 216 | 50.739 |
| 2 | The oral neurokinin-1 antagonist aprepitant for the prevention of chemotherapy-induced nausea and vomiting: A multinational, randomized, double-blind, placebo-controlled trial in patients receiving high-dose cisplatin - The Aprepitant Protocol 052 Study Group | Hesketh P.J | JOURNAL OF CLINICAL ONCOLOGY | 2003 | 165 | 50.739 |
| 3 | Delayed nausea and vomiting continue to reduce patients' quality of life after highly and moderately emetogenic chemotherapy despite antiemetic treatment | Bloechl-Daum Brigitte | JOURNAL OF CLINICAL ONCOLOGY | 2006 | 159 | 50.739 |
| 4 | Drug therapy: Chemotherapy-induced nausea and vomiting | Hesketh Paul J. | NEW ENGLAND JOURNAL OF MEDICINE | 2008 | 148 | 176.082 |
| 5 | Antiemetics: American Society of Clinical Oncology Clinical Practice Guideline Update | Hesketh Paul J. | JOURNAL OF CLINICAL ONCOLOGY | 2017 | 146 | 50.739 |
| 6 | Palonosetron plus dexamethasone versus granisetron plus dexamethasone for prevention of nausea and vomiting during chemotherapy: a double-blind, double-dummy, randomised, comparative phase III trial | Saito, Mitsue | LANCET ONCOLOGY | 2009 | 131 | 54.433 |
| 7 | Addition of the neurokinin 1 receptor antagonist aprepitant to standard antiemetic therapy improves control of chemotherapy-induced nausea and vomiting - Results from a randomized, double-blind, placebo-controlled trial in Latin America | Poli-Bigelli, S | CANCER | 2003 | 127 | 6.921 |
| 8 | Efficacy and tolerability of aprepitant for the prevention of chemotherapy-induced nausea and vomiting in patients with breast cancer after moderately emetogenic chemotherapy | Warr DG | JOURNAL OF CLINICAL ONCOLOGY | 2005 | 120 | 50.739 |
| 9 | The effect of guideline-consistent antiemetic therapy on chemotherapy-induced nausea and vomiting (CINV): the Pan European Emesis Registry (PEER). | Aapro, M. | ANNALS OF ONCOLOGY | 2012 | 119 | 51.769 |
| 10 | Olanzapine for the Prevention of Chemotherapy-Induced Nausea and Vomiting | Navari, Rudolph M. | NEW ENGLAND JOURNAL OF MEDICINE | 2016 | 103 | 176.082 |
